# A Miniaturized Piezoelectric MEMS Accelerometer with Polygon Topological Cantilever Structure

**DOI:** 10.3390/mi13101608

**Published:** 2022-09-27

**Authors:** Chaoxiang Yang, Bohao Hu, Liangyu Lu, Zekai Wang, Wenjuan Liu, Chengliang Sun

**Affiliations:** 1 The Institute of Technological Sciences, Wuhan University, Wuhan 430072, China; 2 Hubei Yangtze Memory Laboratories, Wuhan 430072, China

**Keywords:** piezoelectric MEMS accelerometers, AlN, miniaturized, polygon topological cantilever

## Abstract

This work proposes a miniaturized piezoelectric MEMS accelerometer based on polygonal topology with an area of only 868 × 833 μm^2^. The device consists of six trapezoidal cantilever beams with shorter fixed sides. Meanwhile, a device with larger fixed sides is also designed for comparison. The theoretical and finite element models are established to analyze the effect of the beam′s effective stiffness on the output voltage and natural frequency. As the stiffness of the device decreases, the natural frequency of the device decreases while the output signal increases. The proposed polygonal topology with shorter fixed sides has higher voltage sensitivity than the larger fixed one based on finite element simulations. The piezoelectric accelerometers are fabricated using Cavity-SOI substrates with a core piezoelectric film of aluminum nitride (AlN) of about 928 nm. The fabricated piezoelectric MEMS accelerometers have good linearity (0.99996) at accelerations less than 2 g. The measured natural frequency of the accelerometer with shorter fixed sides is 98 kHz, and the sensitivity, resolution, and minimum detectable signal at 400 Hz are 1.553 mV/g, 1 mg, and 2 mg, respectively. Compared with the traditional trapezoidal cantilever with the same diaphragm area, its output voltage sensitivity is increased by 22.48%.

## 1. Introduction

MEMS accelerometers are key components of inertial measurement units (IMU) and are widely used in inertial navigation applications for consumer intelligent electronic devices and handheld positioning devices [1]. The MEMS accelerometer is developing towards further reduction in size, high resolution, high sensitivity, and high stability. High-performance piezoelectric MEMS accelerometers are widely used in various fields: condition monitoring systems [2,3,4], electronic stability control and airbags in the automotive industry [5], sensors in smart electronic products [6], flight detection [7,8], seismometers for earthquake monitoring [9], Internet of Things [3,10], etc.

Micro accelerometers are generally classified as capacitive, piezoresistive, thermal and resonant types [11]. Compared with other types of accelerometers, piezoelectric MEMS accelerometers have the advantages of high sensitivity, low power consumption, small temperature dependence, and high bandwidth. Piezoelectric-based MEMS devices have shown high energy conversion in sensing elements [12]. Although accelerometers with bulk piezoelectric materials have higher sensitivity, they are bulky and expensive to manufacture, which is not in line with the trend of gradually miniaturizing sensors. Therefore, the MEMS fabrication shows great potential for the miniaturized applications. The micromachined PZT films are still the most widely used piezoelectric material for piezoelectric accelerometers because of their high piezoelectric properties [13,14,15]. However, in recent years, piezoelectric materials have gradually shifted to lead-free materials, so accelerometers based on aluminum nitride (AlN) films have attracted much attention due to the advantages of miniaturization, low manufacturing cost, non-toxicity, and compatibility with CMOS circuits.

Many researchers have recently studied piezoelectric MEMS accelerometers using AlN as piezoelectric material. Ze-Hui Chen et al. proposed an AlN-based MEMS accelerometer with a sensitivity of 1.49 mV/g and a natural frequency of 7.2 kHz [16]. Nidhi Gupta et al. studied the effect of residual stress in AlN (0 0 2) film on the MEMS accelerometer. The presence of residual stress reduces the resonant frequency (up to 14.72%) and bandwidth (up to 27.3%) of the accelerometer [17]. Jian Yang et al. studied an AlN-based resonant MEMS accelerometer that senses acceleration by changes in resonant frequency. The overall structure size is 464 × 650 μm^2^, and the sensing axis sensitivity is 1.11 Hz/g (68.9 ppm/g) [18]. Bohao Hu et al. demonstrated a ScAlN/AlN-based piezoelectric MEMS accelerometer using the trapezoidal with corners (TWC)-shaped cantilevers. It has voltage sensitivity 7.95 mV/g [19]. Liu Yan et al. proposed a triaxial accelerometer based on a ScAlN/AlN composite piezoelectric film, with charge sensitivities along the x, y, and z axes up to ~1.07 pC/g, ~0.66 pC/g, and ~3.35 pC/g [20]. In summary, all these accelerometers consist of a mass and a resonator. In order to improve the sensitivity, a larger mass or a micro leverage structure is used [9]. However, smart sensors have a demand for miniaturized MEMS devices.

In this paper, two types of miniaturized piezoelectric MEMS accelerometers were designed and fabricated on Cavity-SOI substrates using AlN micromachined processes. The piezoelectric accelerometers contain six trapezoidal cantilever beams with an area of 868 × 833 μm^2^. The natural frequency, sensitivity, resolution, and minimum detectable signal of the accelerometer were measured, and the fabricated device exhibited good linearity (0.99996) at accelerations less than 2 g. Compared with the traditional trapezoidal cantilever under the same diaphragm area, the output voltage sensitivity of the proposed piezoelectric MEMS accelerometer is improved by 22.48%.

## 2. Device and Simulation

### 2.1. Structural Design

The proposed piezoelectric MEMS accelerometer comprises six trapezoidal cantilever beams whose short sides are fixed on a central support column to form a polygonal topology. This structure is built on a cavity SOI substrate with a specific shape. After sensing the acceleration, the cantilever beam in the piezoelectric MEMS accelerometer will bend and deform, thereby generating a corresponding electrical signal.

**Figure 1:**
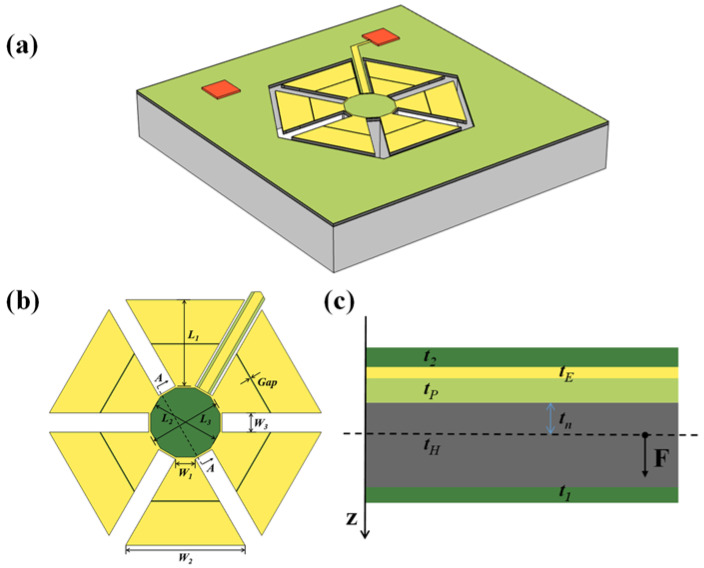
(**a**) Three-dimensional view, (**b**) front view, and (**c**) cross-sectional view of the designed miniaturized piezoelectric MEMS accelerometer with polygonal topology.

The structure diagram is presented in Figure 1, in which Figure 1a–c shows the three-dimensional, front, and cross-sectional views of the designed miniaturized piezoelectric MEMS accelerometer with polygonal topology. The designed piezoelectric MEMS accelerometer consists of six trapezoidal cantilever beams whose free side length is larger than the fixed side length, which causes a greater stress near the fixed side. The theoretical and finite element models are established to analyze the effect of the beam′s effective stiffness on the output voltage and natural frequency. The structure relies on an extra bridge to pull the collected electrical signal onto the signal pads.

The specific structural dimensions of this device are shown in Table 1. The top electrode is very thin compared to other material layers, so the top electrode has little effect on the natural frequency of the structure, and it can be neglected. The first task in determining the stiffness of the beam is to find the neutral axis of the cantilever. The neutral plane is the plane in which the normal force in the x_1-direction is zero if nothing other than a bending moment is applied to the beam.

For a symmetrical beam of uniform material, the neutral axis is the middle of the beam. However, in a composite beam, the effect of the different moduli of each layer must be considered to find the neutral axis [21]:

(1) $$\int_{-t_{n}-t_{P}-t_{2}}^{-t_{n}-t_{P}}\frac{E_{O}}{\rho_{O}}zdz+\int_{-t_{n}-t_{P}}^{-t_{n}}\frac{E_{P}}{\rho_{P}}zdz+\int_{-t_{n}}^{t_{H}-t_{n}}\frac{E_{HDS}}{\rho_{HDS}}zdz+\int_{t_{H}-t_{n}}^{t_{H}-t_{n}+t_{1}}\frac{E_{O}}{\rho_{O}}zdz=0$$

After calculation, the position of the neutral axis is:

(2) $$t_{n}=\frac{\frac{E_{HDS}t_{H}^{2}}{2\rho_{HDS}}-\frac{E_{P}t_{P}^{2}}{2\rho_{P}}+\frac{E_{O}({\left(t_{1}+t_{H}\right)}^{2}-t_{H}^{2})}{2\rho_{O}}-\frac{E_{O}({\left(t_{2}+t_{P}\right)}^{2}-t_{P}^{2})}{2\rho_{O}}}{\frac{E_{HDS}t_{H}}{\rho_{HDS}}+\frac{E_{P}t_{P}}{\rho_{P}}+\frac{E_{O}\left(t_{1}+t_{2}\right)}{\rho_{O}}}$$

where $E_{O}$, $E_{HDS}$, $E_{P}$ and $t_{1}$, $t_{2}$, $t_{H}$, $t_{P}$ are the Young’s modulus and the thickness of the oxide layer, highly dispersible silica layer (HDS), and piezoelectric layer, respectively. The width of the trapezoidal cantilever can be expressed as:

(3) $$W\left(x\right)=\frac{W_{2}-W_{1}}{L}x+W_{1}$$

Since the thin top electrode has little effect on the natural frequency of the structure, it is omitted to simplify the calculation. The equivalent height of the cantilever beams can be considered as [22]:

(4) $$H=t_{1}+t_{2}+t_{P}+t_{H}$$

The equivalent density of the cantilever beams can be considered as:

(5) $$\rho^{*}=\frac{\rho_{O}\left(t_{1}+t_{2}\right)+\rho_{HDS}t_{H}+\rho_{P}t_{P}}{H}$$

where $\rho_{O}$, $\rho_{HDS}$, and $\rho_{P}$ are the density of the oxide layer, HDS layer, and piezoelectric layer. The equivalent Young′s modulus is:

(6) $$E^{*}=\frac{\sum E_{i}t_{i}}{\sum t_{i}}=\frac{E_{O}\left(t_{1}+t_{2}\right)+E_{HDS}t_{H}+E_{P}t_{P}}{H}$$

The quality of the cantilever can be expressed as follows:

(7) $$m=\int_{0}^{L}A\left(x\right)\rho dx$$

where $A\left(x\right)=W\left(x\right)H=\frac{H\left(W_{2}-W_{1}\right)}{L}x+W_{0}H$ means the cross-sectional area of the trapezoidal beam. If $A\left(x\right)=ax+b$, the equivalent mass of the trapezoidal beam is:

(8) $$m^{*}=\int_{0}^{L}\left(ax+b\right)\rho {\left(\frac{3}{2}{\left(\frac{x}{L}\right)}^{2}-\frac{1}{2}{\left(\frac{x}{L}\right)}^{3}\right)}^{2}dx\;=\left(\frac{43}{224}aL+\frac{33}{140}b\right)\rho L\;=\left(\frac{43H\left(W_{2}-W_{1}\right)}{224}+\frac{33HW_{1}}{140}\right)\rho L\;\;\;\;\;\;$$

According to the equation of inertia moments, the equivalent inertia moment of a trapezoidal cantilever can be calculated as Equation (9) [23].

(9) $$I^{*}=\int_{-t_{n}}^{t_{H}-t_{n}}z^{2}Wdz+\frac{E_{O}}{E_{H}}\int_{t_{H}-t_{n}}^{t_{H}-t_{n}+t_{1}}z^{2}Wdz+\frac{E_{P}}{E_{H}}\int_{-t_{n}-t_{P}}^{-t_{n}}z^{2}Wdz+\frac{E_{O}}{E_{H}}\int_{-t_{n}-t_{P}-t_{2}}^{-t_{n}-t_{P}}z^{2}Wdz\;=\frac{W}{3}(t_{n}^{3}+{\left(t_{H}-t_{n}\right)}^{3})+\frac{WE_{P}}{3E_{HDS}}({\left(t_{n}+t_{P}\right)}^{3}-t_{n}^{3})\;+\frac{WE_{O}}{3E_{HDS}}({\left(t_{1}+t_{H}+t_{n}\right)}^{3}-{\left(t_{H}-t_{n}\right)}^{3}-{\left(t_{n}+t_{P}\right)}^{3}+{\left(t_{n}+t_{P}+t_{2}\right)}^{3})$$

Substituting Equations (6) and (8) into Equation (9), we can obtain the natural frequency of a trapezoidal cantilever as shown in Equation (10).

(10) $$\begin{array}{ll} f & =\frac{1}{2\pi }\sqrt{\frac{3E^{*}I^{*}}{m^{*}L^{3}}}\\ & =\frac{1}{2\pi }\sqrt{\frac{WA\left(E_{HDS}\left(t_{n}^{3}+{\left(t_{H}-t_{n}\right)}^{3}\right)+E_{O}B\right)+E_{P}\left({\left(t_{n}+t_{P}\right)}^{3}-t_{n}^{3}\right)}{\left(\frac{43H\left(W_{2}-W_{1}\right)}{224}+\frac{33HW_{1}}{140}\right)\rho HL^{4}E_{HDS}}} \end{array}$$

where $A=(E_{O}\left(t_{1}+t_{2}\right)+E_{HDS}t_{H}+E_{P}t_{P})$, $B={\left(t_{1}+t_{H}+t_{n}\right)}^{3}-{\left(t_{H}-t_{n}\right)}^{3}-{\left(t_{n}+t_{P}\right)}^{3}+{\left(t_{n}+t_{P}+t_{2}\right)}^{3}$.

When the device is subjected to a force F in the +z direction, the beam will bend and deform in the +z direction. After calculation, we can know that $t_{n}>0$ means that the neutral layer of the cantilever is located below the piezoelectric layer. Therefore, the piezoelectric layer will be subjected to tensile stress. The bending moment of the cantilever at position x of the cantilever can be expressed as:

(11) $$M\left(x\right)=F\left(L_{1}-x\right)$$

The induced electric field D generated by the piezoelectric film in the z direction is:

(12) $$D=\frac{g_{31}E_{p}F\left(L_{1}-x\right)\left(t_{n}-z\right)}{WE^{*}I^{*}}$$

where $g_{31}$ is the piezoelectric voltage constant of the piezoelectric material. The induced voltage of the trapezoidal beam can be expressed as [19]:

(13) $$V=\int_{-t_{n}-t_{P}}^{-t_{n}}\frac{g_{31}E_{p}F\left(L_{1}-x\right)\left(t_{n}-z\right)}{WE^{*}I^{*}}dz=\frac{g_{31}E_{p}F\left(L_{1}-x\right)\left(2t_{n}+\frac{t_{P}^{2}}{2}\right)}{\left[\frac{\left(W_{2}-W_{1}\right)x}{L_{1}}+W_{1}\right]E^{*}I^{*}}$$

In conclusion, the voltage calculated by simulation should be the arithmetic mean of the sensing voltage of the effective upper electrode, which can be expressed as:

(14) $$\;\;V_{m}=\frac{1}{L_{4}}\int_{0}^{L_{4}}\frac{g_{31}E_{p}F\left(L_{1}-x\right)\left(2t_{n}+\frac{t_{P}^{2}}{2}\right)}{\left[\frac{\left(W_{2}-W_{1}\right)x}{L_{1}}+W_{1}\right]E^{*}I^{*}}dx\;=\frac{E_{p}FL_{1}g_{31}}{2E^{*}I^{*}{\left(W_{2}-W_{1}\right)}^{2}}\left[\begin{array}{c} \left(t_{P}^{2}+4t_{n})(L_{4}W_{1}-L_{4}W_{2}+L_{1}W_{2}\ln(L_{1}W_{1}-L_{4}(W_{1}-W_{2}))\right)\\ -L_{1}W_{2}\ln(L_{1}W_{1}(t_{P}^{2}+4t_{n})) \end{array}\right]\;\;\;\;$$

where $L_{4}$ is the length of the effective electrode. It can be seen that the output voltage of the trapezoidal cantilever is directly related to the cantilever length $L_{1}$, the fixed end length $W_{1}$, the free end length $W_{2}$, and the effective electrode length $L_{4}$. The proposed device structure is aimed at miniaturization. It aims to provide a piezoelectric MEMS accelerometer that is high-sensitivity, high-linearity, and as miniaturized as possible for future intelligent wearable devices. Therefore, the natural frequency of the device is higher than in other research, which is unavoidable with size reduction.

### 2.2. Finite Element Simulation

**Figure 2:**
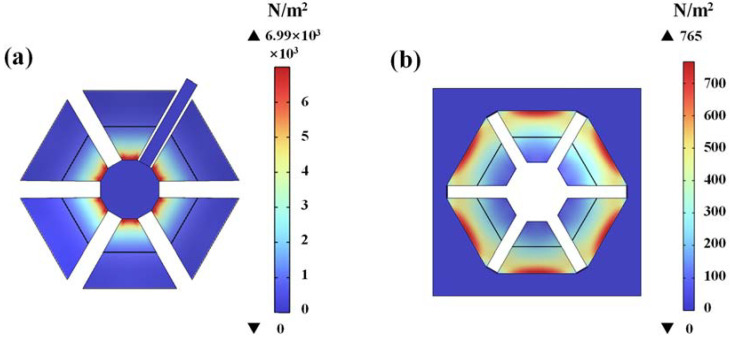
Stress distribution diagrams of (**a**) Type A and (**b**) Type B piezoelectric MEMS accelerometers at 400 Hz.

The proposed piezoelectric MEMS accelerometer is simulated with COMSOL multiphysics coupling software in this work. The cantilevers of the device will bend and deform under a force. Figure 2 shows the stress distribution diagrams of Type A and Type B piezoelectric MEMS accelerometers at 400 Hz. It shows that the normal stress is mainly concentrated near the fixed end area of the cantilever. The stress gradually decreases to zero in the area away from the fixed end of the cantilever. Figure 2a shows that the max stress of Type A is 6.99 × 10^3^ Pa, while that of Type B in Figure 2b is 765 Pa. According to the piezoelectric effect, the induced charges generated by the device are mainly concentrated in the area with high stress. Therefore, the electrode area involved in the electrical connection is concentrated near the fixed end of the cantilever in this work.

The natural frequency of Type A and Type B calculated by Equation (10) is 98.51 kHz and 253.05 kHz, while it is 98.9 kHz and 257.91 kHz simulated in COMSOL software. The theoretical derivation is consistent with the simulation results. The material parameters used in the simulations are shown in Table 2.

## 3. Fabrication and Characterization

### 3.1. Fabrication Process

**Figure 3:**
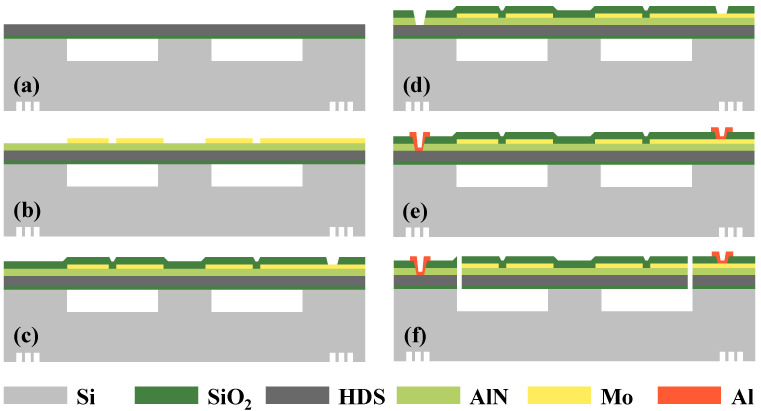
Schematic illustrations of the proposed piezoelectric MEMS accelerometer fabrication flow.

The above devices are fabricated using a five-mask AlN process. The process flow is shown in Figure 3. First, an 8-inch CSOI (cavity silicon-on-insulator) substrate was prepared with thicknesses of 725 μm silicon, 50 μm cavity, 1 μm oxide, and 5.2 μm HDS (highly dispersible silica), as shown in Figure 3a. Figure 3b shows the deposition of 1 μm AlN thin films on HDS, followed by the deposition and patterning of 150 nm thick Mo as electrode material. An oxide layer of 2 μm was then deposited to protect the Mo electrode from oxidation, and patterning was performed to expose the Mo electrode, as shown in Figure 3c. The oxide layer and piezoelectric layer were sequentially etched to expose the HDS layer, as shown in Figure 3d. Figure 3e shows that a 1 μm Al layer as electrical pads were evaporated on top of the oxide layer and patterned using a lift-off process to lead out the electrical connections of the HDS layer and the Mo layer, respectively. Finally, Figure 3f shows that the device layer is etched and released to form the cantilever beams structure.

### 3.2. Morphological Characterization

**Figure 4:**
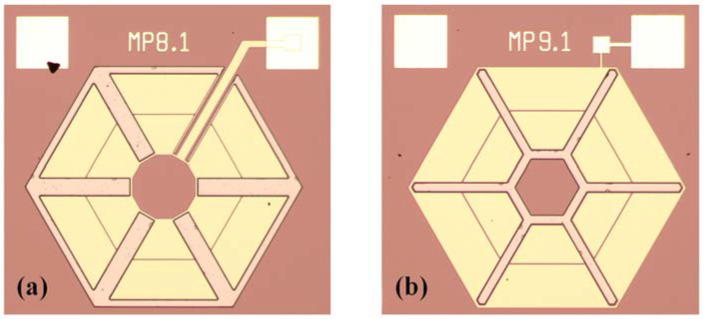
The front view diagram of (**a**) Type A and (**b**) Type B using an optical microscope.

As shown in Figure 4, the fabricated devices are in similar diaphragm areas, and both consist of six cantilevers. Type A has a larger free end for each cantilever beam than that of the fixed end, while the structure of Type B is the opposite. Type B is the opposite of the proposed structure Type A fixing method. In addition, the device and the PCB board are connected through gold wires.

**Figure 5:**
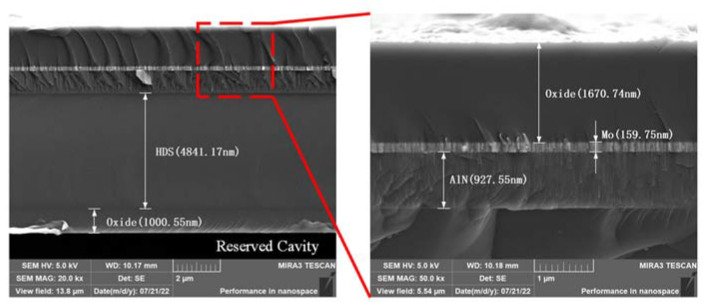
Cross-sectional SEM images of the proposed piezoelectric MEMS accelerometer.

Figure 5 shows the cross-sectional view of the device, of which the measured thickness of the oxide layer above the cavity is about 1001 nm; the thickness of the HDS layer is about 4841 nm; the thickness of the AlN piezoelectric layer is about 928 nm; the thickness of the Mo electrode layer is about 160 nm; and the thickness of the uppermost oxide layer is about 1671 nm. The thickness of the HDS layer and the uppermost oxide layer deviates from the design values since the thickness of the stack deviates during deposition and to some extent during SEM measurement. Therefore, the formula derivation and simulation results need to be corrected according to the actual thickness of the device. The rest of this paper shows the corrected formula derivation and simulation results.

### 3.3. Dynamic Characterization

**Figure 6:**
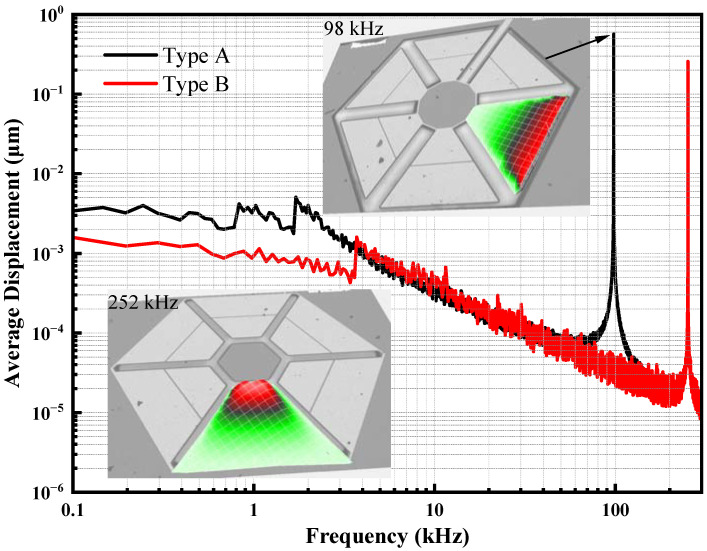
Frequency response measurement results using a Polytec MSA-600 LDV.

The frequency response directly measures the maximum displacement amplitude of the free end of a cantilever beam on a piezoelectric MEMS accelerometer using a PolyTec MSA-600 Laser Doppler Vibrometer (LDV). Figure 6 shows a graph of the displacement of the free end of the cantilever beam versus frequency for a piezoelectric MEMS accelerometer. The measured resonant frequencies of the piezoelectric MEMS accelerometer are 98 kHz and 252 kHz, respectively. The point displacement of Type A and Type B at the resonant frequency in air is 0.565 μm and 0.257 μm, respectively, under a 1-Vpp (peak-to-peak) drive voltage. The static capacitances of the piezoelectric MEMS accelerometer are also measured using E4980A Precision LCR Meter. The measured static capacitances of these two piezoelectric MEMS accelerometers are around 9 pF and 20 pF, respectively, at 400 Hz.

Since the length of the fixed end of Type A is smaller than that of the free end, the effective stiffness of Type A is greatly reduced compared to the Type B, whose fixed end is longer than the free end. The sensitivity of the accelerometer is inversely proportional to its stiffness (provided via support cantilevers) [26]. Therefore, Type A has a lower natural frequency and higher voltage sensitivity than Type B.

## 4. Results and Analysis

### 4.1. Experimental Procedure

**Figure 7:**
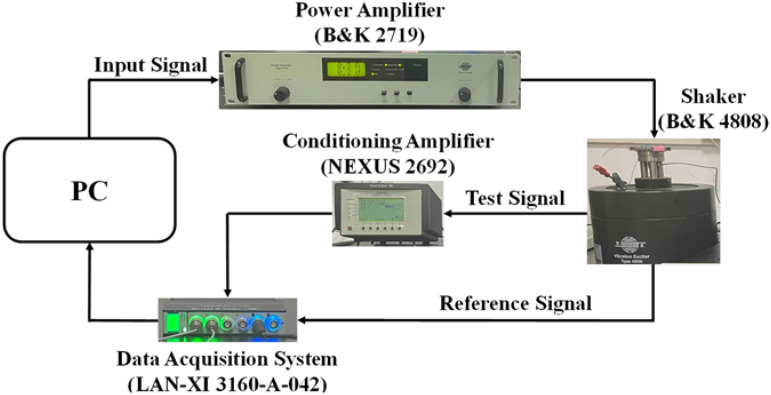
The diagram of the accelerometer vibration experiment setup.

In the experiment setup, a Brüel & Kjær (B&K) 4808 uniaxial shaker is selected as the excitation source, with a maximum force capacity of 112 N within the operating frequency of 5 Hz to 10 kHz [27]. There has a reference accelerometer on the B&K 4808 shaker to measure the vibration acceleration. B&K 2719 power amplifier is used for amplitude amplification. The excitation voltage is controlled by the FULSE software on the PC while amplified by the power amplifier and delivered to the 4808 uniaxial shaker. The experiment setup is shown in Figure 7. Piezoelectric MEMS accelerometer devices are epoxy-glued to the PCB board and then fixed on the B&k 4808 shaker. During the test, the charge signal generated by the piezoelectric MEMS accelerometer is delivered to the conditioning amplifier (NEXUS 2692). After amplification and transformation, the data is sent to the data acquisition system (LAN-XI 3160-A-042) and displayed on the PULSE software.

### 4.2. Results and Discussion

**Figure 8:**
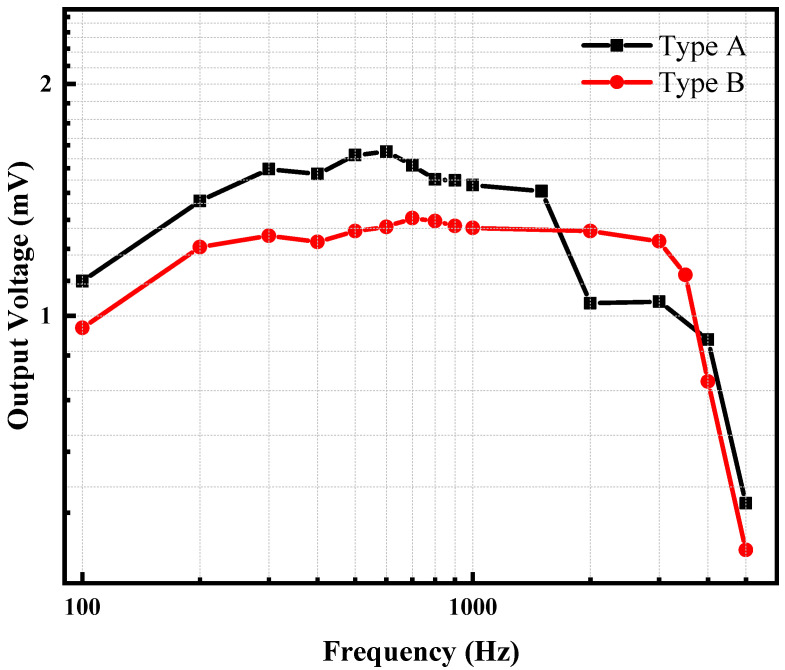
Frequency response curve formed by single frequency sweeps of the experiment system.

The excitation voltage is adjusted on the PULSE software, then amplified by the power amplifier and transmitted to the B&K 4808 shaker to adjust the vibration acceleration of the shaker. The vibration acceleration can be measured by the reference accelerometer and fed back to the PULSE software. Figure 8 shows the frequency response curve of Type A and Type B under 1 g acceleration, and the data of the frequency response curve are based on the test results of multiple frequency points. Type A and Type B have almost the same diaphragm area. The operating frequency range of Type A piezoelectric MEMS accelerometer is about 200 Hz~1.5 kHz, while the operating frequency range of Type B is about 200 Hz~3 kHz. In addition, the experimental results show that Type A piezoelectric MEMS accelerometers have significantly higher output voltage, while Type B has a wider operating frequency range.

**Figure 9:**
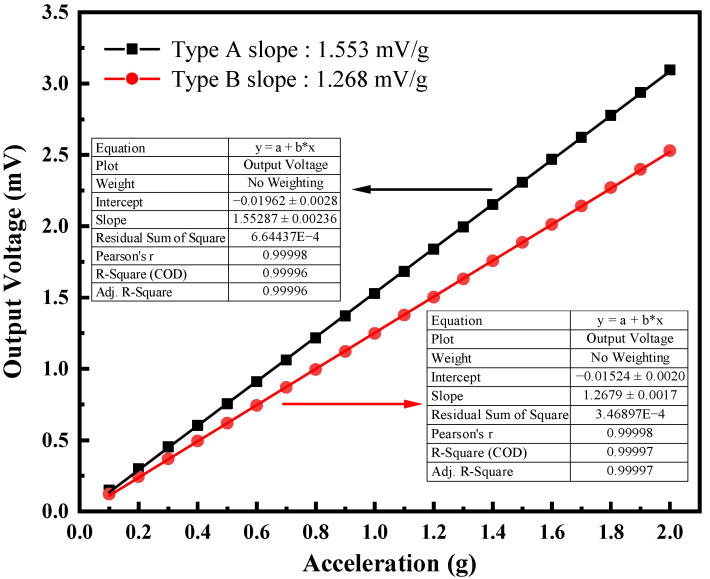
Output voltage vs. acceleration for Type A and Type B piezoelectric MEMS accelerometers to obtain the sensitivity and linearity at 400 Hz.

**Figure 10:**
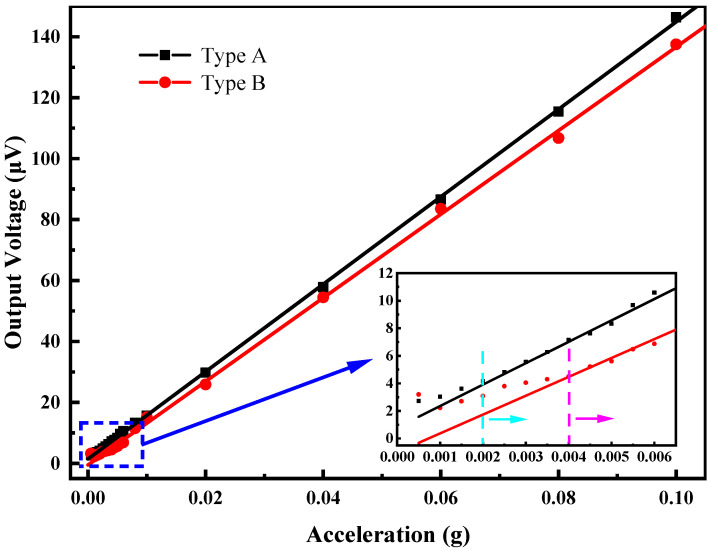
Minimum detectable signal (MDS) of Type A and Type B piezoelectric MEMS accelerometers at 400 Hz.

Figure 9 shows the output voltage of two piezoelectric MEMS accelerometers with acceleration varying from 0.1 g to 2 g at 400 Hz. The slopes of these two fitting curves reflected the sensitivities of Type A and Type B are 1.553 mV/g and 1.268 mV/g, respectively. The output voltage characteristics of these two types achieved good linearity. The output of the piezoelectric MEMS accelerometer no longer exhibits a linear change if the vibration acceleration of the shaker is tiny. As shown in Figure 10, the minimum detectable acceleration of Type A and Type B piezoelectric MEMS accelerometers is 2 mg and 4 mg, respectively. The output of Type A is no longer linear when sensing acceleration is less than 2 mg, while Type B has an obvious deviation below a sensing acceleration of 4 mg.

**Figure 11:**
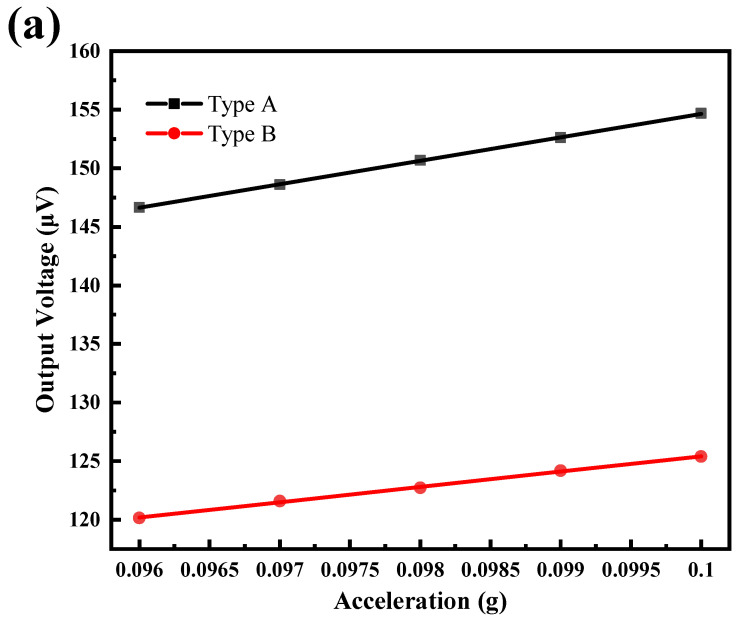

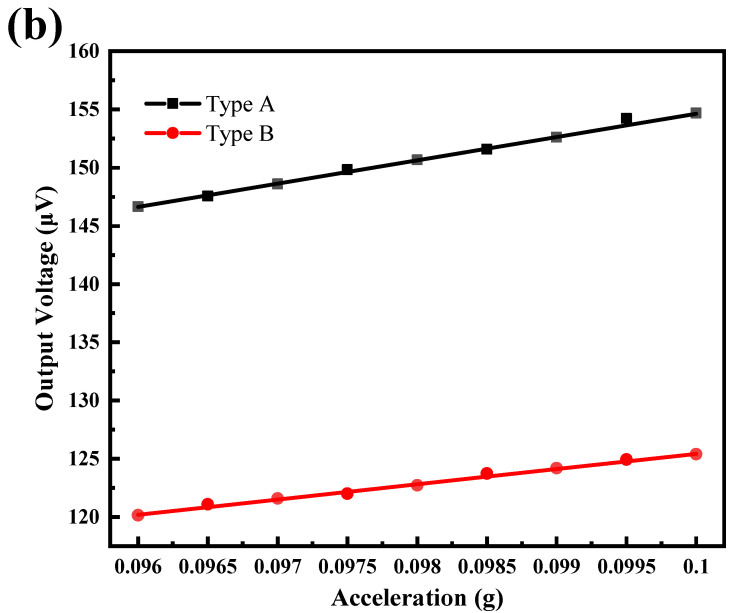
Output voltage vs. acceleration for Type A and Type B piezoelectric accelerometers to obtain the resolution at 400 Hz. Sweep step size of excitation acceleration: (**a**) 1 mg, (**b**) 0.5 mg.

Figure 11 shows the output responses of the two piezoelectric MEMS accelerometers at 400 Hz with an acceleration of around 0.1 g and sweep step size of 1 mg and 0.5 mg. Figure 11a shows that both Types A and B can accurately detect acceleration changes of 1 mg, exhibiting good linearity. Figure 11b shows the partial distortion of the output response at a sweep step size of 0.5 mg. When the scan step size is 0.5 mg, the accelerometer cannot accurately measure the acceleration. Therefore, we calibrated the resolution of these two piezoelectric MEMS accelerometers to 1 mg. In addition, the thermal noise of Type A and Type B accelerometers at 400 Hz and room temperature (T = 300 K) are 841 nV/√Hz and 882.4 nV/√Hz, respectively.

Compared with the work of other research groups in Table 3, the polygon topological structure proposed in this paper can achieve almost the same performance at a much smaller device size. Moreover, the MEMS accelerometer in this work also has good linearity and resolution.

## 5. Conclusions

In this paper, a miniaturized piezoelectric MEMS accelerometer based on polygonal topology with an area of only 868 × 833 μm^2^ was designed, fabricated, and tested. The proposed polygonal topology with shorter fixed sides has higher voltage sensitivity than the larger fixed one based on finite element simulations. The FEM simulation and experimental results verify the influence mechanism of piezoelectric constant, model stiffness, and stress distribution on the proposed accelerometer output electrical response. Polygonal topology with larger free end than fixed end has higher voltage sensitivity of 1.533 mV/g at 400 Hz and a relatively flat response between 200 Hz and 1.5 kHz. The fabricated piezoelectric MEMS accelerometers have good linearity (0.99996) at accelerations less than 2 g. In addition, the minimum detectable acceleration at 400 Hz is 2 mg, and the resolution of the detectable acceleration is 1 mg. Compared with the traditional trapezoidal cantilever under the same diaphragm area, the output voltage sensitivity of the proposed piezoelectric MEMS accelerometer is improved by 22.48%. The proposed piezoelectric MEMS accelerometer shows great potential in high sensitivity and miniaturized accelerometer applications.

## Figures and Tables

**Table 1:** Specific structural dimensions of Type A device.

Symbol	Parameter	Value (μm)
$t_{1}$	Buried oxide thickness	1
$t_{H}$	HDS thickness	5.2
$t_{P}$	AlN thickness	1
$t_{E}$	Mo thickness	0.15
$t_{2}$	Top oxide thickness	1.8
$t_{n}$	Neutral plane position of a simplified model	1.63
$L_{1}$	Trapezoidal beam length	226
$L_{2}$	Distance between opposite beams	182
$L_{3}$	Width of the support column	192
Gap	Break width between electrodes	2
$W_{1}$	Short side width of the trapezoidal beam	50
$W_{2}$	Long side width of the trapezoidal beam	305
$W_{3}$	Spacing distance between beams	54

**Table 2:** Material parameter values in this work.

Material	Piezoelectric Strain Coefficient $d_{31}\;=\;\varepsilon_{r}\varepsilon_{0}g_{31}\left(pC/N\right)$	Young’s Modulus E (GPa)	$\mathrm{Density}\;\rho \;\left(kg/m^{3}\right)$	Poisson’s Ratio v
AlN	−2.65 [24]	244	3300	0.24 [24,25]
Si	-	170	2329	0.28
HDS	-	125.4	2329	0.198
Mo	-	312	10200	0.31
SiO_2_	-	70	2200	0.17

**Table 3:** Summary of main parameters in piezoelectric accelerometers.

Author	C. C. Hindrichsen et al. [28]	Z. H. Chen et al. [16]	B. H. HU et al. [19]	This Work
Piezoelectric material	PZT	AlN	ScAlN/AlN	AlN
Device size [mm^2^]	7.84	63.62	14.25	0.723
Sensitivity	0.31 mV/g (2 kHz)	1.49 mV/g (7.2 kHz)	7.95 mV/g (400 Hz)	1.533 mV/g (400 Hz)
Resonance frequency [kHz]	11	7.2	1.29	98
Device structure	Seismic mass suspended by four beams	Circular membrane	TWC membrane	Polygon topological cantilevers
Compatible with CMOS process	No	Yes	Yes	Yes

## Data Availability

Not applicable.
